# Prognostic factors of esophageal perforation and rupture leading to mortality: a retrospective study

**DOI:** 10.1186/s13019-021-01680-y

**Published:** 2021-10-09

**Authors:** Jong Duk Kim

**Affiliations:** Department of Cardiothoracic Surgery, School of Medicine, Gyeonsang National University, Gyeongsang National University Hospital, Jin-Ju, 79 Gangnam-ro, Jinju-si, Gyeongsangnam-do 52727 Republic of Korea

**Keywords:** Esophageal perforation, Esophageal rupture, Fish bone, Retrospective

## Abstract

**Background:**

Esophageal perforation and rupture (EPR) is a serious, potentially life-threatening condition. However, no treatment methods have been established, and data concerning factors affecting mortality are limited. This report presents the prognostic factors of mortality in EPR based on experience in the management of such patients.

**Methods:**

For this retrospective analysis, 79 patients diagnosed as having EPR between 2006 and 2016 and managed at Gyeongsang National University Hospital were examined. The management method was determined in accordance with the location and size of the EPR, laboratory findings, and radiological findings. Thirty-nine patients were treated with surgery; and 40, with nonsurgical management.

**Results:**

The most common cause of EPR was foreign body (fish bone or meat bone), followed by vomiting, iatrogenic causes, and trauma. Thirty-nine patients underwent primary repair of EPR, of whom 4 patients died. Forty patients underwent nonsurgical management, of whom 3 patients died. The remaining patients were discharged. Mortality correlated with the size of the EPR (> 25 mm) and the segmented neutrophil count percentage (> 86.5%) in the white blood cell test and differential.

**Conclusions:**

The mortality risk was increased when the EPR size and the segmented neutrophil count percentage in the white blood cell test and differential was high. Delayed diagnosis, which was considered an important predictive factor in previous investigations, was not statistically significant in this study.

*Trial registration***:** Not applicable.

## Background

Although esophageal perforation and rupture (EPR) occurs rarely, it is a life-threatening condition that leads to mortality if not treated appropriately. EPR has various causes, especially traumatic and non-traumatic factors. EPR caused by trauma is divided into penetrating injury and blunt trauma. EPR due to penetrating injury is mainly due to gunshot wounds or penetration of foreign bodies, and occurs in < 1% of the total EPR cases [1]. In the case of non-traumatic EPR, the cause is divided into iatrogenic and non-iatrogenic. Iatrogenic causes often occur after endoscopic intervention and are the most common causes [2, 3]. Spontaneous esophageal perforation, also known as Boerhaave syndrome, is reported in 15% of all EPR cases [4]. If EPR is not managed properly, it can lead to localized infections such as mediastinitis, pneumonia, empyema, as well as systemic infections that can lead to mortality. Management of EPR depends on the cause, location, and size of the EPR, and the presence of accompanying symptoms. Spontaneous esophageal perforation is managed by primary repair of the damaged site or insertion of a self-expanded stent. In the case of EPR without pleural effusion or with a small size, nonsurgical management can be applied [5]. The recent advances in management, total parenteral nutrition, antibiotics, and intensive care unit management have led to nonsurgical management of EPR [6]. Despite the recent accumulation of treatment experience and advances in medical technology, the results of EPR treatment have not met expectations, with mortality rates still reaching 4–20%, although other studies have shown slightly different results [7, 8]. Owing to the anatomical structure of the esophagus, unlike other gastrointestinal tract organs in the abdominal cavity, the resistance to distortion is relatively low, which is likely to cause damage due to distortion by mass effect. EPR can lead to severe infective conditions such as empyema, pneumonia, and mediastinitis by gastric content or food from oral intake, which increases mortality. This study was aimed at identifying factors that affect the high mortality of EPR and improving the treatment of EPR.

## Methods

This retrospective study was conducted in 79 patients diagnosed as having EPR at Gyeongsang National University Hospital between January 2006 and December 2016. Of the patients, 52 were male and 27 were female, with a mean age of 64 years (range 30–87 years). EPR was diagnosed on the basis of radiological findings and esophagogastroduodenoscopy (EGD). If a clear esophageal rupture could be observed on chest computed tomography, EGD for EPR confirmation was not performed, but EGD for confirmation was performed if a radiological finding of pleural effusion and pneumomediastinum with symptoms, including chest pain, fever, dyspnea, and subcutaneous emphysema, was suspected. After EPR diagnosis, surgical or nonsurgical management was applied depending on the location and size of the EPR.

### Statistical analyses

Continuous variables were presented as means, ranges, and standard deviations. Categorical variables were presented as frequencies with the associated percentages. All the statistical analyses were performed using the IBM SPSS version 22.0 software (IBM Corp. Armonk, NY, USA).

### Management

**Fig. 1:**
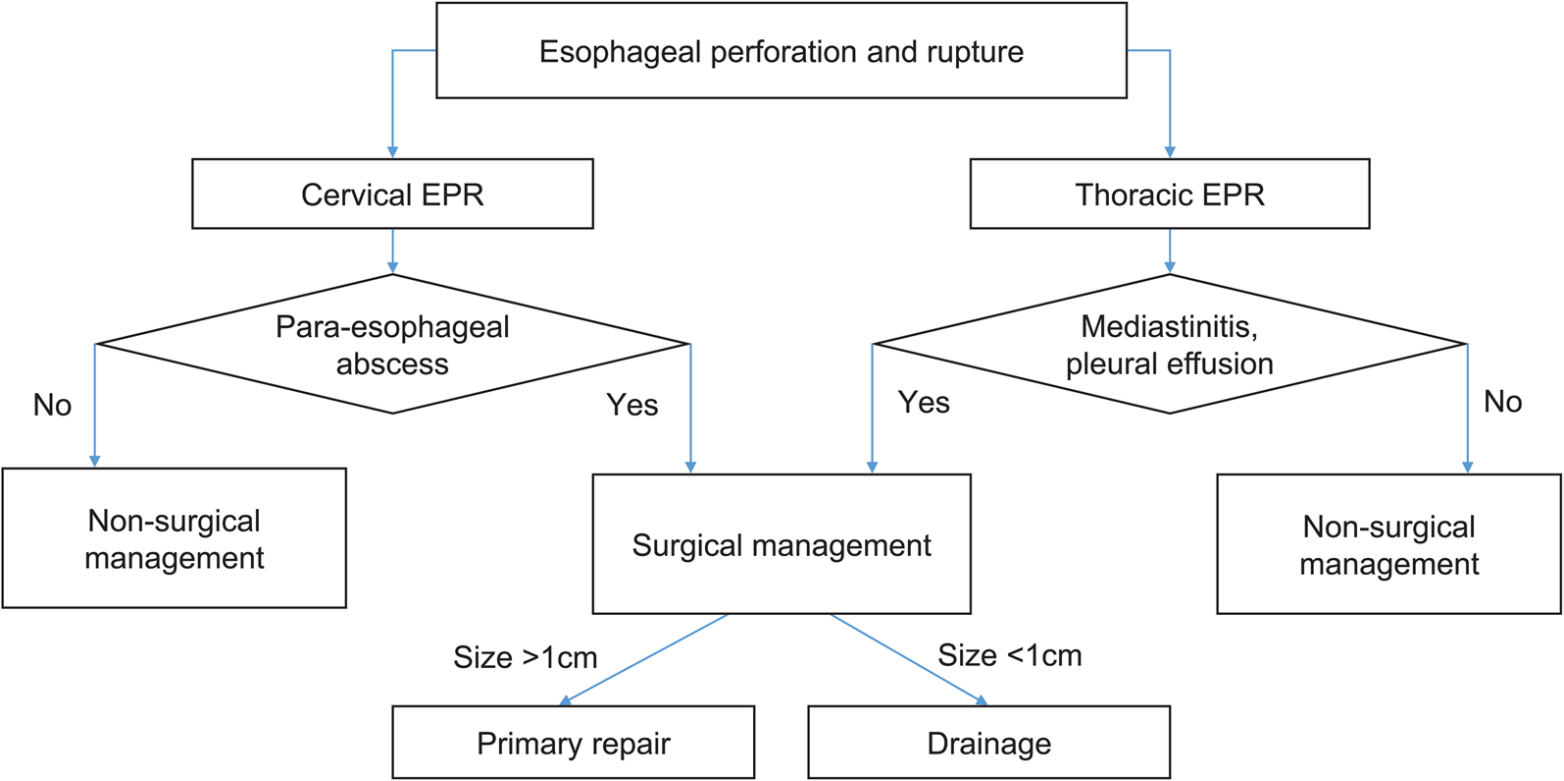
Management of esophageal perforation and rupture (EPR)

The size and location of the EPR identified on EGD and the presence or absence of para-esophageal abscess and pleural effusion determined the treatment method (Fig. 1).

In the case of cervical EPR, nonsurgical treatment with nil per os (NPO) and total parenteral nutrition (TPN) was applied for cases without a para-esophageal abscess, whereas surgical treatment was considered for cases with para-esophageal abscess. If the cervical EPR is smaller than 1 cm, it was exposed through esophageal dissection and then drainage with Penrose or Jackson-Pratt drain for the para-esophageal abscess was performed. If the cervical EPR is > 1 cm, a primary repair was performed with a double-layer suture. After the primary repair, drainage was applied using either a Penrose or Jackson-Pratt drain. In the case of thoracic EPR, similar to determining the treatment direction of cervical EPR, nonsurgical treatment with NPO and TPN was performed for cases without a para-esophageal abscess and pleural effusion. In addition, surgical treatment was performed if a para-esophageal abscess was observed around the EPR or pleural effusion was identified in the radiological findings.

If the size of the thoracic EPR was < 1 cm, the EPR was exposed through a partial esophageal dissection. Moreover, if the size was > 1 cm, a primary repair was performed. In the case of surgical treatment, a large-bore 32-Fr chest tube was inserted.

### Ethical considerations

This study was approved by the Institutional Review Board of the Gyeongsang University College of Medicine (IRB number: 2017-08-008-002). All study participants provided written informed consent for participation. All study procedures were carries out in accordance with the principles in Declaration of Helsinki (1964 and its later amendments). This article does not disclose any personally identifiable data of any of the participants in any form. Hence, consent for publication is not applicable here.

## Results

The most common cause of EPR was foreign body ingestion, accounting for 50 of the 79 patients. In the foreign body cases, fish bone was the most common cause; vomiting and iatrogenic causes were the second and third most common causes. Seven patients died, and all the other patients were discharged from the hospital routinely after an average of 20 days of hospital treatment (Table 1). The thorax was the most common location, followed by the cervical and esophago-gastric junction. The mean size of the EPR was 19.54 mm but showed a slight variation according to location. EPRs with a thoracic location were larger because of the role of the Boerhaave syndrome (Table 2).

**Table 1:** Causes of esophageal perforation and rupture

	Surgical group	Nonsurgical group
Foreign body	20	30
Fish bone	15	26
Other bone	3	2
Denture	1	0
Piece of glass	1	0
Aluminum foil	0	2
Spontaneous (vomiting)	15	7
Iatrogenic	3	2
Trauma	1	0
Previous esophageal disease (achalasia rupture)	1	0
Hospital day	30.53	11.54
Mortality	4	3
Total	40	39

**Table 2:** Location of the esophageal perforation and rupture

Esophageal location^a^	Number (%)	Size (mm)
Cervical	13 (16.5)	12.69
Thoracic		
Upper	12 (15.2)	16.58
Middle	19 (24.0)	12.79
Lower	18 (22.8)	24.06
EG junction	17 (21.5)	29.54
Total	79	19.54

Esophageal locations^a^: cervical esophagus, 15–18 cm from the incisor; upper thoracic esophagus, 19–24 cm below the incisor; middle thoracic esophagus, 25–32 cm below the incisor; lower thoracic esophagus, 33–37 cm below the incisor; EG junction, 38–40 cm below the incisor

### Surgical management

Among 39 patients, 3 died of multiple-organ failure secondary to exacerbation of mediastinitis and one died of acute respiratory distress syndrome secondary to a worsening postoperative pneumonia. The mean time from surgery to death was 22 days (range 7–35 days). The most important postoperative complication was anastomotic leakage, which occurred in 14 patients. One of the patients underwent reoperation of the leakage and was discharged from the hospital after 15 days of reoperation. The other 13 patients were discharged from the hospital after the initial surgery, with a mean hospital stay of 10 days.

### Nonsurgical management

Of the 40 patients, 3 needed surgery but rejected the procedure; thus, surgery was not performed. Two patients needed surgery for EPR with the Boerhaave syndrome at a size of 20 and 40 mm, respectively. The patients who needed surgery but did not undergo the procedure died of multiple-organ failure due to sepsis on the 27th and seventh hospital days, respectively. The other patient had a 20-mm-sized EPR with Boerhaave syndrome was discharged from the hospital after 20 days of nonsurgical management. With the exception of the 3 patients who died, the other 37 patients had a Clavien-Dindo scale score of ≥ 2 and were all discharged without further adverse events [9].

### Prognostic factors

Factors influencing the mortality of patients with EPR were assumed to be the degree of systemic infection at the time of diagnosis and the size of EPR. Segmented neutrophil count percentage performed at the time of EPR diagnosis was used to evaluate the degree of influence on the patient's systemic infection. Since there was a large difference in the number of dead and surviving patients, an analysis method using the receiver operating characteristic curve was used to evaluate the effect on death.

**Fig. 2:**
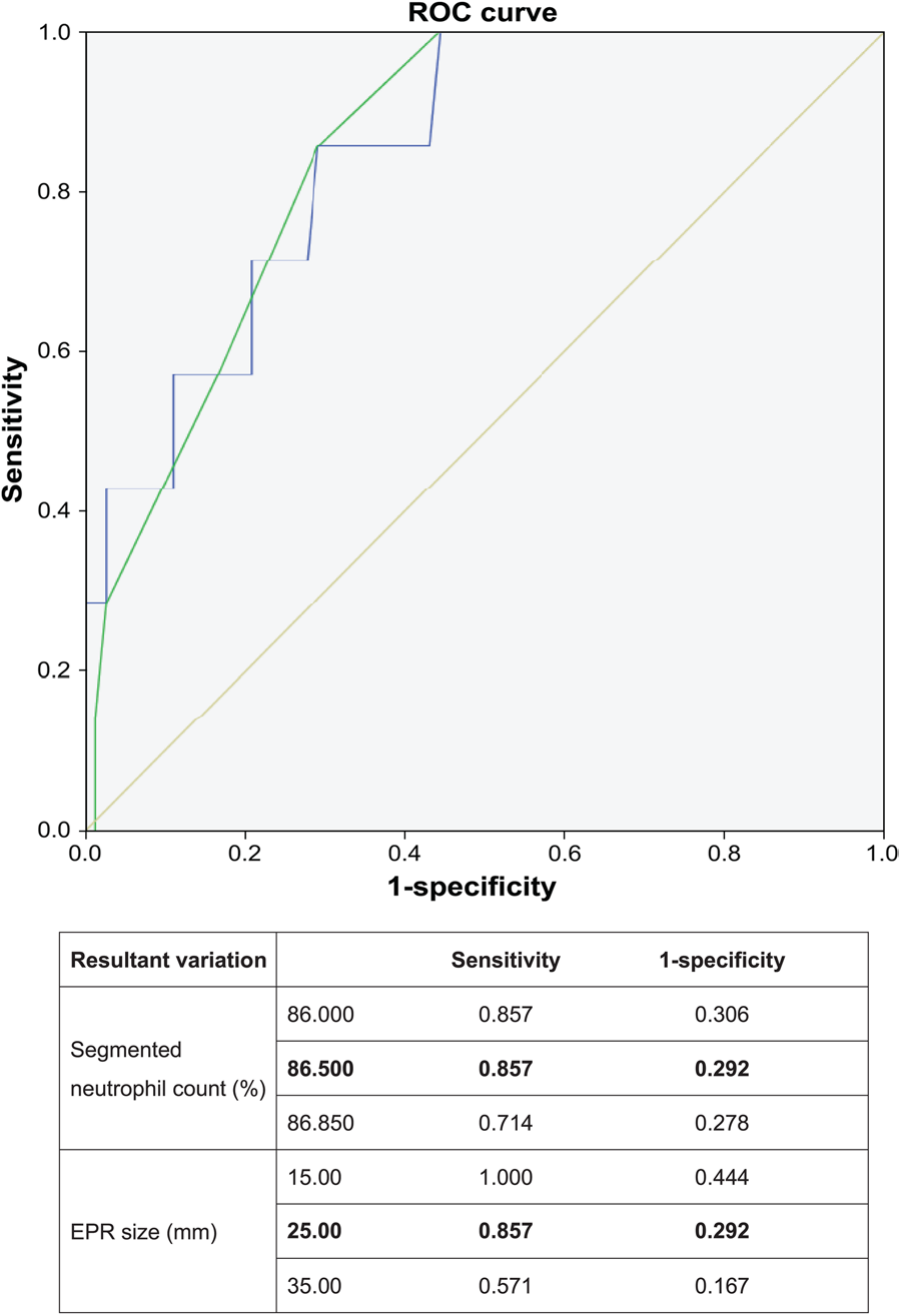
Receiver-operating curve analysis of mortality

In the analysis using the receiver operating characteristic curve, in the case of segmented neutrophil count percentage, the value of sensitivity at 86.5% was 0.857, and in the case of EPR size, the value of sensitivity at 25 mm was 0.857. Based on these results, it was found that the mortality rate increases when the segmented neutrophil count percentage is ≥ 86.5% and when the SIZE of EPR is > 25 mm at the time of EPR diagnosis (Fig. 2).

## Discussion

The first case of spontaneous esophageal rupture (Boerhaave syndrome) was reported 300 years ago. Until 1947, no successful treatment was available. In 1947, Olson and Clagett attempted surgical treatment of EPR; several years later, a successful treatment approach was achieved [10]. The goals of treatment for EPR are to control systemic infection and early oral intake. The development of systemic infection has a strong negative impact on EPR prognosis.

The management of EPR has always been challenging because when not properly treated, EPR can lead to patient death [11]. In addition, the relatively low incidence of EPR makes it difficult for medical providers, including physicians, to gain sufficient experience in providing appropriate treatment for the condition [12, 13].

In recent decades, endoscopic examination of digestive organs and transesophageal echography for cardiovascular disease evaluation have had great impacts on the early detection and improved the outcomes of digestive system and heart diseases [14]. However, the incidence of iatrogenic esophageal perforation as assessed using endoscopic examinations has also increased in proportion to the incidence of EPR [2, 15]. Recent studies have suggested that iatrogenic causes are the most common etiology of EPR [16, 17]. However, the most common cause of EPR in the present study was ingestion of fish bone. This is considered to occur because of a combination of ingestion of relatively small and sharp seaweed fish bones and the carelessness of the patient while eating fish. EPR due to fish bone has several characteristics. First, it can usually be diagnosed rapidly. The main reason for EPR treatment failure is delayed diagnosis and intervention [15, 18]. This is because the symptoms of EPR are usually nonspecific, such as chest discomfort, orthopnea, fever, and dysphagia [8, 19–21]. Therefore, time is required to differentiate EPR from coronary artery disease, aortic dissection, pericardial effusion, and pneumonia. In the absence of rapid treatment following the occurrence of EPR, gastric and oral content leakages leading to mediastinitis and other infections are more likely to occur, which can increase mortality and morbidity.

According to previous reports, the most important prognostic factor in EPR is the time from onset to treatment. In most previous studies, patients treated within 24 h of presentation showed better outcomes than those with more delayed treatment [3, 9, 19, 22, 23].

Esophageal perforation due to fish bone can present with a clear picture of the time of development and the location of the EPR as compared with other causes because acute pain with dysphagia is prominent in most patients.

The reason why early diagnosis and management for EPR shows a good prognosis is thought to be a way to prevent the rapid progression of mediastinitis, which may occur after EPR, to sepsis. Unfortunately, this study did not show the results of early diagnosis and management with good prognosis. Perhaps this is due to the relatively small-sized study in single institution, It is judged to have shown no significant statistical results.

The second characteristic of EPR due to fish bone ingestion is that the size of the EPR is smaller than that associated with Boerhaave syndrome or iatrogenic causes. The size of the EPR is an important feature that affects prognosis. Therefore, esophageal perforation due to fish bone, in comparison with other causes, seems to have a good clinical prognosis (Table 3).

**Table 3:** Characteristics of esophageal perforation and rupture (EPR) according to etiology

Cause of EPR	No. of cases	Size (mm), mean	Time to treatment (days)	Hospital stay duration	Mortality, n (%)
Fish bone	41	10.80	2.06	15.07	2 (4.87%)
Boerhaave syndrome	22	33.64	2.32	29.82	3 (13.6%)
Iatrogenic	5	16.80	3.7	19.4	1 (20%)
Other	11	25.7	2.61	28	1 (9.1%)
Total	79				7

In previous studies, the mortality of EPR was reported to range from 20 to 30%, but in our study, the mortality rate was 10% (8/79), which is relatively low [8, 24]. This is due to the high proportion of cases involving small EPRs due to fish bone. With the development of radiological diagnostic techniques, the size and location of the esophageal perforation and the presence of complications such as mediastinitis or abscess can be diagnosed more accurately than ever before. The clinical outcome of EPR is improving owing to the advancements in TPN, antibiotic therapy, and accumulation of treatment experience with EPR. However, some patients still require surgical intervention, and death can still occur without adequate treatment. EPR warrants further study as a serious, potentially life-threatening condition.

The present author aimed to identify the factors that affect the outcome of EPR in addition to the timing of treatment initiation. Therefore, this study was conducted under the assumption that the EPR size and initial inflammatory values (white blood cell [WBC] count, segmented neutrophil count percentage, C-reactive protein [CRP] level) would affect the outcome of EPR. Among these factors, segmented neutrophil count percentage and EPR size were found to be statistically significantly associated with the outcome of EPR. Although the WBC and CRP values of the patients who died were higher than those of the patients who survived, no statistically significant differences were found.

This study had limitations such as its retrospective design and relatively small number of patients. Further study will require additional consideration to overcome these limitations.

## Conclusions

Ingestion of fish bone is one of the most common causes of EPR, and this etiology was associated with a significantly better prognosis. EPR due to fish bone correlated with a relatively small size and was associated with rapid diagnosis. The mortality risk was increased when the EPR size and the segmented neutrophil count percentage in the white blood cell test and differential was high.

## Data Availability

The data that support the findings of this study are available from the corresponding author upon reasonable request.

## Abbreviations

CRP: C-reactive protein
EGD: Esophagogastroduodenoscopy
EPR: Esophageal perforation and rupture
NPO: Nil per os
TPN: Total parenteral nutrition
WBC: White blood cell
